# Prognostic Impact of the Systemic Immune-Inflammation Index According to Concurrent Chemotherapy Backbone in Stage III Non-Small Cell Lung Cancer

**DOI:** 10.3390/curroncol33070407

**Published:** 2026-07-09

**Authors:** Aykut Demirkıran, Murat Araz, Melek Karakurt Eryılmaz, Mustafa Karaağaç, Muhammed Muhiddin Er, Berrin Benli Yavuz, Mehmet Artaç

**Affiliations:** 1 Department of Medical Oncology, Konya City Hospital, 42090 Konya, Türkiye; 2 Department of Medical Oncology, Necmettin Erbakan University, 42080 Konya, Türkiye; 3 Department of Medical Oncology, Medical Park Hospital, 55200 Samsun, Türkiye; 4 Department of Radiation Oncology, Necmettin Erbakan University, 42080 Konya, Türkiye

**Keywords:** unresectable stage III NSCLC, definitive chemoradiotherapy, systemic immune-inflammation index, prognostic factor, concurrent chemotherapy, survival

## Abstract

Clinical outcomes remain heterogeneous among patients with unresectable stage III non-small cell lung cancer treated with definitive chemoradiotherapy. We evaluated the prognostic value of the systemic immune-inflammation index (SII) and explored survival outcomes according to the concurrent chemotherapy backbone in this setting. This retrospective study included 101 patients treated with definitive chemoradiotherapy before consolidation durvalumab became standard practice. Baseline SII was calculated using routine blood counts. Overall survival (OS) and progression-free survival (PFS) were analyzed using the Kaplan–Meier method and Cox proportional hazards models. With a median follow-up of 40.6 months, patients with low SII had significantly longer OS (26.1 vs. 10.9 months, *p* = 0.004) and PFS (15.6 vs. 7.2 months, *p* = 0.018) than those with high SII. Elevated SII remained independently associated with inferior OS in multivariable analysis (HR 1.99, 95% CI 1.21–3.27, *p* = 0.007). Although survival was numerically longer with cisplatin/etoposide than with carboplatin/paclitaxel, the difference was not statistically significant. Subgroup analysis according to SII level and chemotherapy regimen showed the poorest outcomes among patients with high SII receiving carboplatin/paclitaxel. These findings support the prognostic value of SII and suggest a potential role for risk stratification in patients with unresectable stage III NSCLC undergoing definitive chemoradiotherapy in the pre-durvalumab era.

## Simple Summary

Patients with unresectable stage III non-small cell lung cancer are commonly treated with definitive chemoradiotherapy. Despite receiving similar treatments, survival outcomes vary considerably among patients. Blood-based inflammatory markers obtained from routine laboratory tests may help identify individuals at higher risk of poor outcomes. In this study, we investigated the systemic immune-inflammation index (SII), a marker calculated from routine blood counts, in patients treated with definitive chemoradiotherapy. Patients with elevated SII had shorter survival and earlier disease progression than those with lower SII values. We also observed that survival outcomes differed according to the combination of baseline SII and the concurrent chemotherapy regimen. These findings suggest that SII may serve as a simple and readily available biomarker for risk stratification in patients with unresectable stage III NSCLC.

## 1. Introduction

Lung cancer continues to pose a major global health burden and remains the most common cause of cancer-related death worldwide. Recent estimates indicate that approximately 2.5 million new cases and 1.8 million deaths were recorded in 2022 [1]. Among patients diagnosed with non-small cell lung cancer (NSCLC), nearly one-third present with stage III disease. This stage comprises a clinically heterogeneous population with marked differences in tumor extent, nodal involvement, and survival outcomes [2]. For patients with unresectable stage III NSCLC, definitive concurrent chemoradiotherapy (CRT) remains the standard treatment approach and has consistently demonstrated superior survival compared with sequential chemoradiotherapy [3,4]. The management of unresectable stage III NSCLC has evolved substantially over the past decade. Improvements in radiotherapy planning and delivery techniques have enhanced treatment precision while minimizing radiation exposure to surrounding normal tissues. More recently, the introduction of consolidation immunotherapy following definitive chemoradiotherapy has further improved long-term outcomes and established a new treatment paradigm for this patient population [4,5]. Nevertheless, recurrence rates remain considerable, and survival outcomes continue to vary markedly among patients receiving similar treatments. This clinical heterogeneity highlights the need for robust prognostic biomarkers that can assist in risk stratification and facilitate more individualized treatment approaches [5].

Despite advances in radiotherapy techniques and systemic treatment strategies, outcomes after definitive chemoradiotherapy remain heterogeneous. This variability has prompted ongoing efforts to identify prognostic markers that can improve risk stratification and help guide clinical management [5]. In recent years, inflammation-based biomarkers have attracted increasing attention because systemic inflammation is closely linked to multiple processes involved in cancer progression, including tumor proliferation, angiogenesis, metastasis, and immune escape [6].

Cancer-related inflammation plays a central role in tumor initiation, progression, and resistance to therapy. Neutrophils, platelets, cytokines, and other inflammatory mediators contribute to angiogenesis, tumor cell proliferation, and metastatic dissemination, while simultaneously suppressing effective antitumor immune responses. Conversely, lymphocytes are essential components of immune surveillance and are associated with improved cancer control. Consequently, biomarkers that reflect the balance between systemic inflammation and host immunity have emerged as attractive prognostic tools in oncology [6]. Among these, composite indices derived from routine blood counts have gained particular interest because they are inexpensive, readily available, and easily applicable in daily clinical practice [7].

The systemic immune-inflammation index (SII), derived from peripheral neutrophil, platelet, and lymphocyte counts, integrates markers of both tumor-related inflammation and host immune status [7]. Elevated SII has been associated with adverse clinical outcomes across a variety of malignancies, including surgically treated, metastatic, and locally advanced NSCLC populations [8,9,10]. However, the prognostic significance of SII may vary according to disease stage, treatment strategy, and patient characteristics, and its role in patients undergoing definitive chemoradiotherapy remains incompletely defined.

In routine clinical practice, cisplatin/etoposide and carboplatin/paclitaxel are the most commonly used concurrent chemotherapy regimens during definitive chemoradiotherapy. Although these regimens have been compared in previous studies, little is known about whether the prognostic impact of systemic inflammation differs according to the concurrent chemotherapy backbone [11,12]. In particular, the interaction between baseline inflammatory status and chemotherapy regimen in patients undergoing definitive chemoradiotherapy has not been well characterized.

Therefore, this study aimed to investigate the prognostic value of baseline SII in patients with unresectable stage III NSCLC undergoing definitive chemoradiotherapy. In addition, we examined survival outcomes according to concurrent chemotherapy regimen and explored the combined impact of inflammatory status and chemotherapy backbone on clinical outcomes.

## 2. Materials and Methods

### 2.1. Study Design and Patient Population

This retrospective single-center study included consecutive patients with histologically confirmed unresectable stage III non-small cell lung cancer (NSCLC) who received definitive chemoradiotherapy between January 2015 and December 2021. Patients with metastatic disease at diagnosis, prior thoracic radiotherapy, incomplete baseline laboratory data, or insufficient follow-up information were excluded. Only patients with stage IIIA, IIIB, or IIIC disease according to the eighth edition of the TNM classification were eligible for inclusion. A total of 101 patients met the study criteria and were included in the final analysis. The study was conducted in accordance with the Declaration of Helsinki and approved by the Ethics Committee of Necmettin Erbakan University Meram Faculty of Medicine (approval number: 2021/3516).

### 2.2. Treatment Characteristics

All patients received definitive thoracic radiotherapy with concurrent chemotherapy. Radiotherapy was delivered according to institutional protocols, with a median prescribed dose of 64 Gy. Concurrent chemotherapy consisted of either cisplatin/etoposide (EP) or weekly carboplatin/paclitaxel (CP), selected according to physician discretion, patient characteristics, performance status, and comorbidities. Selected patients also received induction chemotherapy before definitive chemoradiotherapy. No patient received consolidation durvalumab. Therefore, the cohort represents a pre-durvalumab treatment population, allowing evaluation of the prognostic impact of inflammatory biomarkers without the potential confounding effects of consolidation immunotherapy.

### 2.3. Data Collection and Inflammatory Biomarkers

Clinical, demographic, pathological, treatment-related, and laboratory data were retrospectively collected from institutional electronic medical records. Baseline laboratory parameters obtained within 14 days before initiation of chemoradiotherapy were used for biomarker calculations. For patients who received induction chemotherapy, these laboratory values were obtained after completion of induction treatment and immediately before the start of definitive chemoradiotherapy. The systemic immune-inflammation index (SII) was calculated as platelet count × neutrophil count/lymphocyte count. In addition to SII, other commonly used inflammation-based biomarkers, including the neutrophil-to-lymphocyte ratio (NLR), platelet-to-lymphocyte ratio (PLR), and prognostic nutritional index (PNI), were also assessed. Patients were classified into low- and high-SII groups using the median baseline SII value (902), which yielded approximately equal-sized groups for survival analyses.

### 2.4. Survival Assessment

Overall survival (OS) was defined as the interval between the first day of definitive chemoradiotherapy and death from any cause or last follow-up. Progression-free survival (PFS) was calculated from the start of definitive chemoradiotherapy until objective disease progression, death from any cause, or the date of the most recent disease assessment in patients without an event. The duration of follow-up was determined using the reverse Kaplan–Meier method.

### 2.5. Statistical Analysis

Statistical analyses were conducted using IBM SPSS Statistics version 20.0 (IBM Corp., Armonk, NY, USA). Continuous variables are reported as median values with corresponding ranges, while categorical data are presented as absolute numbers and percentages. Overall survival (OS) and progression-free survival (PFS) distributions were evaluated using the Kaplan–Meier method and compared by means of the log-rank test. Follow-up duration was estimated according to the reverse Kaplan–Meier technique. Variables with *p* values below 0.10 in univariable analyses and clinically relevant covariates were entered into the multivariable Cox proportional hazards model. Because SII, NLR, and PLR are mathematically related inflammatory indices, only SII was retained in the multivariable model to avoid multicollinearity. Hazard ratios (HRs) together with their 95% confidence intervals (CIs) were reported. All statistical tests were two-tailed, and a *p* value below 0.05 was considered indicative of statistical significance.

## 3. Results

### 3.1. Patient Characteristics

A total of 101 patients with unresectable stage III NSCLC were included in the study. Baseline demographic, clinical, and treatment characteristics are summarized in Table 1.

The median age was 64 years. Most patients were male (97.0%), and squamous cell carcinoma was the predominant histological subtype (73.3%). ECOG performance status was 0–1 in 83.2% of patients. Concurrent chemotherapy consisted of cisplatin/etoposide (EP) in 33 patients (32.7%) and carboplatin/paclitaxel (CP) in 68 patients (67.3%). Induction chemotherapy was administered to 48 patients (47.5%). Stage IIIB disease was the most common stage at diagnosis (56.4%), followed by stage IIIA (25.7%) and stage IIIC (17.8%). The median follow-up duration, estimated using the reverse Kaplan–Meier method, was 40.6 months.

### 3.2. Survival Outcomes

For the entire cohort, median overall survival (OS) was 16.2 months, and median progression-free survival (PFS) was 9.7 months.

### 3.3. Prognostic Impact of Systemic Immune-Inflammation Index

**Figure 1:**
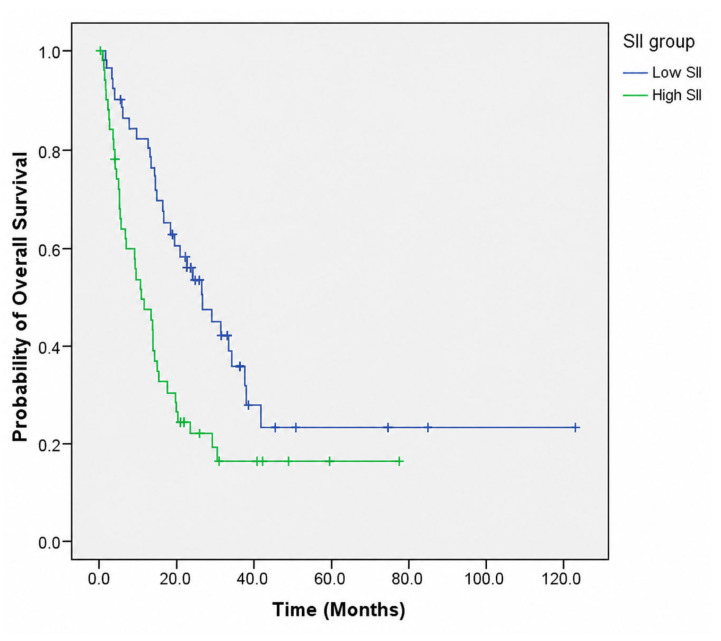
Kaplan–Meier overall survival curves according to systemic immune-inflammation index groups. Patients with high SII demonstrated significantly inferior overall survival compared with patients with low SII (median overall survival: 10.9 vs. 26.1 months; log-rank *p* = 0.004).

**Figure 2:**
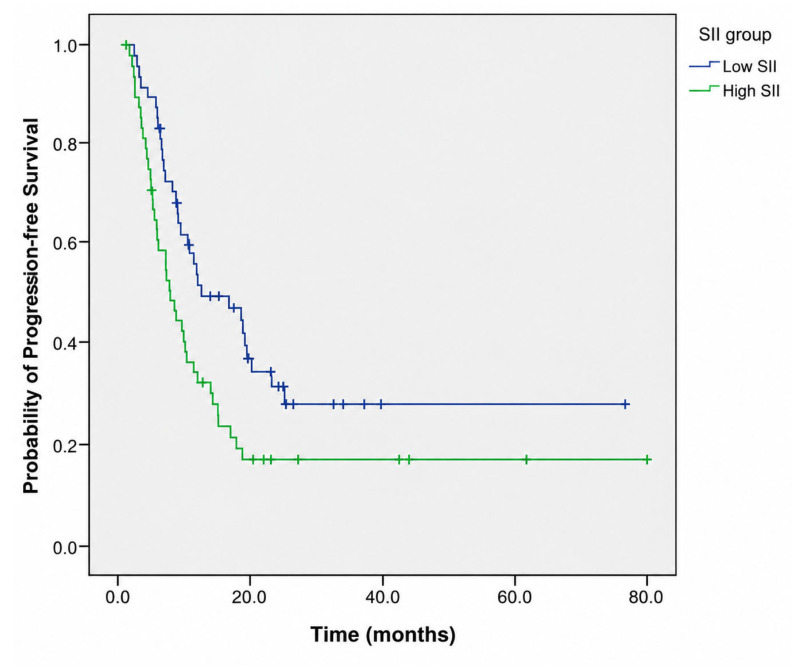
Kaplan–Meier progression-free survival curves according to systemic immune-inflammation index groups. Patients with high SII experienced significantly shorter progression-free survival than patients with low SII (median progression-free survival: 7.2 vs. 15.6 months; log-rank *p* = 0.018).

Patients with low SII demonstrated significantly longer survival outcomes than patients with high SII. Median overall survival was 26.1 months (95% CI, 17.2–34.9) in the low-SII group and 10.9 months (95% CI, 5.8–16.0) in the high-SII group (log-rank *p* = 0.004; Figure 1). Median progression-free survival was 15.6 months (95% CI, 8.2–23.1) in the low-SII group and 7.2 months (95% CI, 4.5–9.8) in the high-SII group (log-rank *p* = 0.018; Figure 2).

Survival outcomes according to inflammatory biomarker groups are presented in Table 2.

### 3.4. Survival According to Concurrent Chemotherapy Backbone

**Figure 3:**
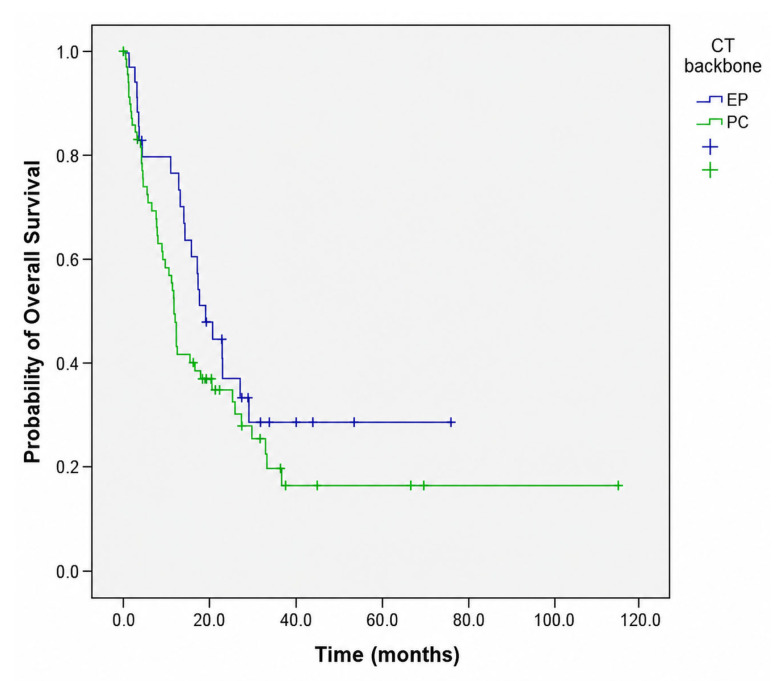
Kaplan–Meier overall survival curves according to concurrent chemotherapy backbone. Patients receiving cisplatin/etoposide demonstrated numerically longer overall survival than those receiving carboplatin/paclitaxel (median overall survival: 21.9 vs. 13.9 months), although the difference did not reach statistical significance (log-rank *p* = 0.163).

Patients treated with EP demonstrated numerically longer survival than those treated with CP. Median overall survival was 21.9 months (95% CI, 16.8–27.1) in patients treated with cisplatin/etoposide and 13.9 months (95% CI, 12.3–15.5) in those treated with carboplatin/paclitaxel. Although a numerical advantage was observed for cisplatin/etoposide, the difference was not statistically significant (log-rank *p* = 0.163; Figure 3).

### 3.5. Integrated Analysis of Systemic Immune-Inflammation Index and Chemotherapy Backbone

**Figure 4:**
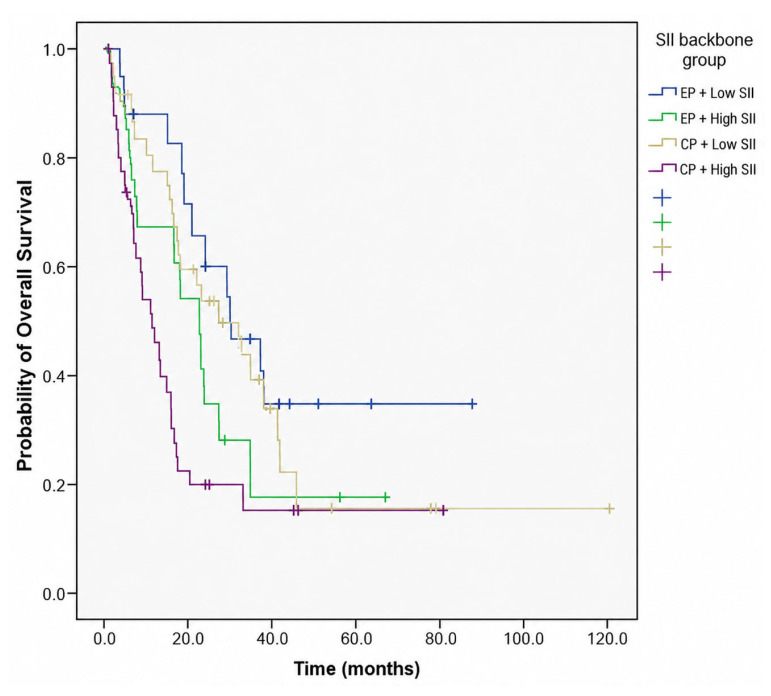
Kaplan–Meier overall survival curves according to integrated systemic immune-inflammation index and chemotherapy backbone groups. Patients with low SII demonstrated more favorable survival outcomes irrespective of chemotherapy regimen. The poorest outcomes were observed in patients with high SII receiving carboplatin/paclitaxel (median overall survival, 9.4 months), whereas the most favorable outcomes were observed in patients with low SII receiving cisplatin/etoposide (median overall survival, 26.1 months). Overall survival distributions differed significantly among the four groups (log-rank *p* = 0.012).

When patients were stratified according to both baseline SII and chemotherapy backbone, significant differences in overall survival were observed among the four groups (log-rank *p* = 0.012; Figure 4). The most favorable outcomes were observed in the EP + low SII group (median overall survival, 26.1 months), whereas the poorest outcomes were observed in the CP + high SII group (median overall survival, 9.4 months). Intermediate outcomes were observed in the EP + high SII (median overall survival, 19.8 months) and CP + low SII (median overall survival, 23.4 months) groups.

### 3.6. Multivariable Analysis

Variables with *p* values below 0.10 in univariable analyses and clinically relevant covariates, including age, disease stage, ECOG performance status, and chemotherapy backbone, were entered into the multivariable Cox regression model. Because SII, NLR, and PLR are mathematically related inflammatory indices, only SII was retained in the multivariable model to avoid multicollinearity. After adjustment for age, disease stage, ECOG performance status, and chemotherapy backbone, high SII remained independently associated with inferior overall survival (HR 1.99, 95% CI 1.21–3.27, *p* = 0.007). No other variable retained statistical significance in the adjusted model. To further evaluate whether the prognostic effect of SII differed according to concurrent chemotherapy backbone, an additional Cox proportional hazards model including an interaction term (SII × chemotherapy regimen) was performed. No statistically significant interaction was observed (HR 1.06, 95% CI 0.38–2.93, *p* = 0.917). Therefore, the subgroup findings should be interpreted as exploratory and hypothesis-generating rather than evidence of a statistically significant interaction. Multivariable Cox regression results are presented in Table 3 and Table 4.

## 4. Discussion

The present study identified baseline SII as an independent prognostic factor in patients with unresectable stage III NSCLC treated with definitive chemoradiotherapy. Elevated SII was associated with significantly shorter overall survival after adjustment for age, disease stage, ECOG performance status, and concurrent chemotherapy regimen (HR 1.99, 95% CI 1.21–3.27, *p* = 0.007). Although patients treated with cisplatin/etoposide experienced longer survival than those receiving carboplatin/paclitaxel, the difference was not statistically significant. Exploratory subgroup analyses demonstrated different survival patterns according to baseline SII and chemotherapy backbone, with patients receiving carboplatin/paclitaxel and having high baseline SII showing the least favorable outcomes. However, the formal interaction analysis did not demonstrate a statistically significant interaction between baseline SII and chemotherapy regimen.

An exploratory analysis incorporating both baseline SII and concurrent chemotherapy regimen revealed marked differences in clinical outcomes across patient subgroups. Patients with high SII who received carboplatin/paclitaxel had the shortest survival, whereas those with low SII treated with cisplatin/etoposide showed the most favorable outcomes. Although these observations should be interpreted cautiously given the retrospective nature of the study and the limited sample size, they reflect different survival patterns observed in exploratory subgroup analyses rather than evidence of a statistically significant interaction between baseline SII and chemotherapy backbone.

The biological association between systemic inflammation and adverse cancer outcomes has been widely documented. Neutrophils and platelets contribute to tumor progression through multiple mechanisms, including promotion of angiogenesis, facilitation of tumor cell dissemination, and suppression of antitumor immune responses. Conversely, lymphocytes are essential mediators of immune surveillance and tumor control [6]. By incorporating neutrophil, platelet, and lymphocyte counts into a single metric, SII may provide a broader reflection of the balance between tumor-promoting inflammatory activity and host immune competence than individual hematologic parameters alone [7,8].

Previous studies have consistently linked elevated SII with unfavorable outcomes in patients with NSCLC. Meta-analyses have shown that high pretreatment SII is associated with shorter survival across a range of disease stages and treatment settings [8,9,10,13]. The results of the present study are consistent with this body of evidence and extend these observations to patients with unresectable stage III NSCLC treated with definitive chemoradiotherapy. In contrast to many earlier reports that included mixed-stage or mixed-treatment populations, our analysis was conducted in a more uniform clinical setting, allowing evaluation of the prognostic value of SII among patients receiving definitive chemoradiotherapy. Compared with other inflammation-based biomarkers such as the neutrophil-to-lymphocyte ratio (NLR) and platelet-to-lymphocyte ratio (PLR), SII incorporates three distinct hematologic parameters and may therefore provide a more comprehensive assessment of the balance between systemic inflammation and host immunity [7,8]. Several studies have suggested that SII demonstrates superior prognostic performance compared with individual inflammatory indices in a variety of solid tumors [8,13]. Because SII integrates information from neutrophils, platelets, and lymphocytes into a single composite marker, it may better reflect the complex biological processes underlying tumor progression and treatment resistance [7,8]. The independent association between elevated SII and inferior overall survival observed in the present study further supports the clinical relevance of this biomarker in patients undergoing definitive chemoradiotherapy.

A distinctive feature of this study was the evaluation of SII in the context of concurrent chemotherapy regimen. Cisplatin/etoposide and carboplatin/paclitaxel remain the most commonly used chemotherapy backbones during definitive chemoradiotherapy, yet comparative studies have reported mixed results [11,12]. Although overall survival was numerically longer among patients treated with cisplatin/etoposide, the difference did not reach statistical significance. This finding should be interpreted in light of the limited sample size and the retrospective, non-randomized design of the study. Nevertheless, exploratory subgroup analyses demonstrated different survival patterns according to baseline SII and chemotherapy backbone. Patients with high SII receiving carboplatin/paclitaxel had the least favorable outcomes, whereas patients with low SII showed comparatively longer survival irrespective of the chemotherapy regimen administered. These findings should be interpreted as exploratory and hypothesis-generating.

Exploratory subgroup analyses demonstrated different survival patterns according to baseline SII and concurrent chemotherapy backbone. However, these findings should be interpreted cautiously. Patients with elevated inflammatory burden may represent a subgroup with more aggressive disease biology, impaired antitumor immune responses, and an increased risk of disease progression. In addition, the poorer outcomes observed among patients with high SII receiving carboplatin/paclitaxel may partly reflect treatment-selection bias. In routine clinical practice, carboplatin-based chemoradiotherapy is frequently preferred for patients with poorer performance status, greater comorbidity burden, or reduced tolerance for cisplatin. Therefore, both patient-related factors and biological mechanisms associated with systemic inflammation may have contributed to the observed differences between treatment groups. Although the underlying mechanisms remain incompletely understood, the present findings highlight the need for further studies investigating the relationship between systemic inflammation, treatment response, and survival in patients undergoing definitive chemoradiotherapy [6,7,8]. Furthermore, formal interaction analysis did not demonstrate a statistically significant interaction between baseline SII and concurrent chemotherapy backbone. Therefore, the observed subgroup differences should be interpreted as exploratory and hypothesis-generating and should not be considered evidence of a statistically significant interaction between baseline inflammatory status and chemotherapy backbone.

An additional feature of this study is that all patients were treated in the pre-immunotherapy era, before consolidation durvalumab became standard practice. This allowed assessment of the prognostic value of SII in a relatively uniform treatment setting, without the influence of immune checkpoint inhibitor therapy. Although treatment paradigms for stage III NSCLC have evolved substantially with the incorporation of immunotherapy, identification of prognostic biomarkers remains clinically important [4,5]. The present findings may help inform future studies evaluating the role of inflammatory biomarkers in the contemporary treatment era. Future prospective studies should also investigate whether baseline SII is associated with completion of consolidation durvalumab and whether it retains its prognostic value in patients receiving consolidation immunotherapy. From a clinical perspective, SII offers several practical advantages. It can be calculated easily from routine complete blood count parameters without additional cost or specialized testing. Therefore, SII may represent an attractive tool for everyday clinical decision-making, particularly in institutions where access to advanced molecular or immunologic biomarkers is limited [7,8]. Although SII should not be considered a substitute for established clinical prognostic factors, it may provide complementary information that helps identify patients at increased risk of poor outcomes. Future studies should investigate whether integrating SII with clinical, radiologic, and molecular variables could improve prognostic models for patients with unresectable stage III NSCLC. In addition, future studies should evaluate whether serial changes in SII during and after chemoradiotherapy provide additional prognostic information beyond baseline measurements.

Several limitations of this study should be considered. First, the retrospective design introduces the potential for selection bias, and treatment allocation was not randomized. The choice of concurrent chemotherapy regimen was based on physician discretion according to individual patient characteristics, including performance status, comorbidities, and anticipated treatment tolerance. Therefore, residual baseline differences between the EP and CP groups cannot be excluded. Second, this was a single-center study with a relatively limited sample size, particularly for subgroup analyses. Third, inflammatory biomarkers were assessed only at baseline, and changes during treatment were not evaluated. In addition, several potentially relevant clinical variables, including weight loss, smoking history, baseline albumin, radiation dose intensity, treatment completion rate, and molecular profiling data were not consistently available because of the retrospective nature of the study and therefore could not be incorporated into the multivariable analyses. Furthermore, none of the patients received consolidation durvalumab, which may limit the applicability of these findings to contemporary treatment settings. Finally, the predominantly male and squamous cell carcinoma population may limit the generalizability of our findings to broader populations with unresectable stage III NSCLC. Accordingly, the observed subgroup findings should be regarded as exploratory and hypothesis-generating and require validation in larger prospective cohorts.

Our findings require validation in larger prospective studies, particularly in the era of consolidation durvalumab. Future research should determine whether inflammatory biomarkers such as SII provide prognostic information beyond established clinical factors and remain informative in contemporary treatment settings. In addition, serial assessment of SII during and after chemoradiotherapy may provide incremental prognostic information beyond baseline measurements and should be investigated in future prospective studies.

## 5. Conclusions

In this retrospective cohort of patients with unresectable stage III NSCLC treated with definitive chemoradiotherapy, elevated baseline systemic immune-inflammation index (SII) was independently associated with inferior overall and progression-free survival. Exploratory subgroup analyses demonstrated different survival patterns according to baseline SII and concurrent chemotherapy backbone, with the least favorable outcomes observed among patients with high SII receiving carboplatin/paclitaxel. However, formal interaction analysis did not demonstrate a statistically significant interaction between baseline SII and chemotherapy backbone, and these subgroup findings should therefore be considered exploratory and hypothesis-generating. These findings support the prognostic value of SII in patients undergoing definitive chemoradiotherapy. Prospective validation is warranted, particularly in the contemporary era of consolidation immunotherapy.

## Figures and Tables

**Table 1:** Baseline characteristics of the study population (n = 101).

Characteristic	Value
Age, median (range), years	64 (39–82)
Sex, n (%)	
Male	98 (97.0)
Female	3 (3.0)
ECOG Performance Status, n (%)	
0–1	84 (83.2)
≥2	17 (16.8)
Histology, n (%)	
Squamous cell carcinoma	74 (73.3)
Adenocarcinoma	18 (17.8)
Other NSCLC	9 (8.9)
Stage, n (%)	
IIIA	26 (25.7)
IIIB	57 (56.4)
IIIC	18 (17.8)
Concurrent chemotherapy	
Cisplatin/Etoposide (EP)	33 (32.7)
Carboplatin/Paclitaxel (CP)	68 (67.3)
Induction chemotherapy	
Yes	48 (47.5)
No	53 (52.5)
SII Group	
Low SII	50 (49.5)
High SII	51 (50.5)

Abbreviations: ECOG, Eastern Cooperative Oncology Group; EP, cisplatin/etoposide; CP, carboplatin/paclitaxel; NSCLC, non-small cell lung cancer; SII, systemic immune-inflammation index.

**Table 2:** Survival outcomes according to systemic immune-inflammation index (SII) and concurrent chemotherapy backbone.

Variable	n	Median OS (Months)	*p* Value	Median PFS (Months)	*p* Value
SII Group					
Low SII	50	26.1	0.004	15.6	0.018
High SII	51	10.9		7.2	
Concurrent chemotherapy					
Cisplatin/Etoposide (EP)	33	21.9	0.163	14.3	0.208
Carboplatin/Paclitaxel (CP)	68	13.9		7.8	
Integrated Analysis					
EP + Low SII	18	26.1		18.4	
EP + High SII	15	19.8		13.2	
CP + Low SII	32	23.4		11.0	
CP + High SII	36	9.4	0.012 *	6.6	0.069

* Log-rank test comparing all four integrated groups. Abbreviations: OS, overall survival; PFS, progression-free survival; SII, systemic immune-inflammation index; EP, cisplatin/etoposide; CP, carboplatin/paclitaxel.

**Table 3:** Univariable Cox regression analysis for overall survival.

Variable	HR	95% CI	*p* Value
High SII	1.98	1.24–3.16	0.004
ECOG ≥ 2	2.01	1.13–3.57	0.017
NLR	1.10	1.02–1.20	0.018
PLR	1.002	1.000–1.004	0.068
Age	1.02	0.99–1.05	0.140
Carboplatin/Paclitaxel vs. Cisplatin/Etoposide	1.43	0.86–2.36	0.165
Induction Chemotherapy	0.81	0.51–1.28	0.360
Stage (IIIA–IIIC)	1.15	0.81–1.64	0.421
Squamous vs. Non-squamous Histology	0.84	0.53–1.32	0.441

Abbreviations: HR, hazard ratio; CI, confidence interval; ECOG, Eastern Cooperative Oncology Group; SII, systemic immune-inflammation index; NLR, neutrophil-to-lymphocyte ratio; PLR, platelet-to-lymphocyte ratio.

**Table 4:** Multivariable Cox regression analysis for overall survival.

Variable	HR	95% CI	*p* Value
ECOG ≥ 2	1.26	0.64–2.47	0.507
Age	1.02	0.99–1.05	0.227
Stage	1.16	0.81–1.65	0.424
Carboplatin/Paclitaxel vs. Cisplatin/Etoposide	1.26	0.72–2.19	0.422
High SII	1.99	1.21–3.27	0.007

Abbreviations: HR, hazard ratio; CI, confidence interval; SII, systemic immune-inflammation index.

## Data Availability

The data presented in this study are available upon request from the corresponding author due to institutional and patient privacy restrictions.

## Abbreviations

The following abbreviations are used in this manuscript:

CI	Confidence Interval
CP	Carboplatin/Paclitaxel
CRT	Chemoradiotherapy
ECOG	Eastern Cooperative Oncology Group
EP	Cisplatin/Etoposide
HR	Hazard Ratio
NLR	Neutrophil-to-Lymphocyte Ratio
NSCLC	Non-Small Cell Lung Cancer
OS	Overall Survival
PFS	Progression-Free Survival
PLR	Platelet-to-Lymphocyte Ratio
PNI	Prognostic Nutritional Index
SII	Systemic Immune-Inflammation Index
TNM	Tumor, Node, Metastasis
